# FOXP3 splice variant expression in males and females in healthy populations and in kidney transplant recipients

**DOI:** 10.1038/s41598-024-62149-1

**Published:** 2024-05-27

**Authors:** Qais W. Saleh, Afsaneh Mohammadnejad, Martin Tepel

**Affiliations:** 1 Department of Nephrology, Odense University Hospital, J. B. Winsløws Vej 4, 5000 Odense, Denmark; 2 Cardiovascular and Renal Research, Department of Molecular Medicine, University of Southern Denmark, J.B. Winsløws Vej 21.3, 5000 Odense C, Denmark; 3 Epidemiology, Biostatistics and Biodemography, Department of Public Health, University of Southern Denmark, J.B. Winsløws Vej 9 B, 5000 Odense C, Denmark

**Keywords:** RNA, Kidney, Renal replacement therapy, Cell biology, Immunology, Biomarkers, Nephrology

## Abstract

The forkhead box P3 (FOXP3) transcript is essential for tolerance of alloantigens. Here, we describe the expression of FOXP3 mRNA variants in healthy females and males, and in kidney transplant recipients (KTR). We measured FOXP3 in peripheral blood mononuclear cells from healthy kidney donors (N = 101), and in blood from KTRs (N = 248) before and after transplantation. FOXP3 was measured with quantitative polymerase chain reaction, and differentiated between pre-mature mRNA FOXP3, Total mature FOXP3, FOXP3 in which exon two is spliced, and full length FOXP3. We found similar levels of FOXP3 in healthy female and male kidney donors. We confirmed this result in a publicly available cohort (N = 33) of healthy individuals (GSE97475). Homogenously, female and male KTR FOXP3 levels were similar pre-transplantation, one day post-transplantation and 29 days post-transplantation. This may suggest that kidney transplantation and related immunosuppressive treatments do not influence FOXP3 expression differently in females and males. Finally, fold difference analysis revealed that KTRs express lower levels of mature FOXP3 and higher levels of pre-mature FOXP3 mRNA pre-transplant compared to healthy individuals. This finding may suggest higher pre-mRNA synthesis, lower pre-mRNA degradation, lower spliceosome efficiency or higher degradation of mature FOXP3 mRNA in kidney transplant candidates.

## Introduction

Biological sex plays an essential role in the immunologic tolerance of auto- and alloantigens. This is evident as the female and male immune systems are influenced differently by genetic, hormonal, and environmental factors^1^. In kidney transplant recipients (KTRs), where tolerance for the kidney allograft is a pre-requisite for kidney allograft survival, reports have indicated that young females have a higher risk of graft loss compared to males of a similar age^2–4^. This disparity in graft survival has been explained partly by a higher pre-transplant sensitization to human leukocyte antigens in females^3,5^. However, the underlying molecular basis is not fully understood, and might be elucidated by differences in the female and male immune systems.

The master gene regulator of regulatory T cells, forkhead box P3 (FOXP3), is essential for immune tolerance of auto- and alloantigens^6^. In humans, it is expressed as two major variants^7^. Out of 11 coding exons, ten coding exons are expressed in the most common FOXP3 splice variant, in which exon two is spliced (FOXP3d2). In the second major variant, all coding exons are expressed (FOXP3fl)^7^. The expression of both major FOXP3 variants are essential for the normal function of T regulatory cells^8–10^, and failure or imbalance of FOXP3 splice variant expression leads to severe and multi-organ auto-immune syndromes^6,8^. In KTRs, studies have shown that higher blood FOXP3 mRNA levels are associated with freedom from chronic rejection and better long-term graft function^11^. Furthermore, immunosuppressive medicines have been shown to alter regulatory T cell function and numbers, and consequently also alter FOXP3 levels^11–14^.

The relationship between biological sex, age, and their effects on FOXP3 expression have recently received attention. Two recent studies have measured and compared expression levels of females and males with conflicting results. Singh et. al. reported that healthy males have a 2–threefold increase in FOXP3 expression compared to healthy females, and that FOXP3 expression is affected by sex-hormones^15^. Robinson et. al. report that males have higher absolute numbers of regulatory T cells, but that FOXP3 mRNA expression levels were similar to those in females^16^. Potential differences in FOXP3 splice variant expression between healthy females and males have not been explored. Furthermore, potential sex-based expression of FOXP3 expression has not been explored in KTRs. We aimed to describe FOXP3 mRNA splice variant expression in healthy females and males, and the effects of age on this expression. Furthermore, we also aimed to describe FOXP3 mRNA expression in female and male KTRs, and the effects of age on FOXP3 expression levels.

## Results

### Baseline characteristics

We included 101 healthy kidney donors from the MoMoTx cohort, 60 (59%) of which were females and 41 (41%) were males. The median age of the healthy kidney donors was 51 years [IQR 41 to 59], 51 years [IQR 42 to 59] in females and 47 years [IQR 40 to 59] in males, p > 0.90. The healthy validation dataset included data from 33 healthy hepatitis B vaccine recipients, 20 (60%) of which were females and 13 (40%) were males. The median age in the healthy validation group was 26 years [IQR 19 to 35], 27 years [IQR 22 to 41] in females and 21 years [IQR 19 to 29] in males, p = 0.65.

**Figure 1:**
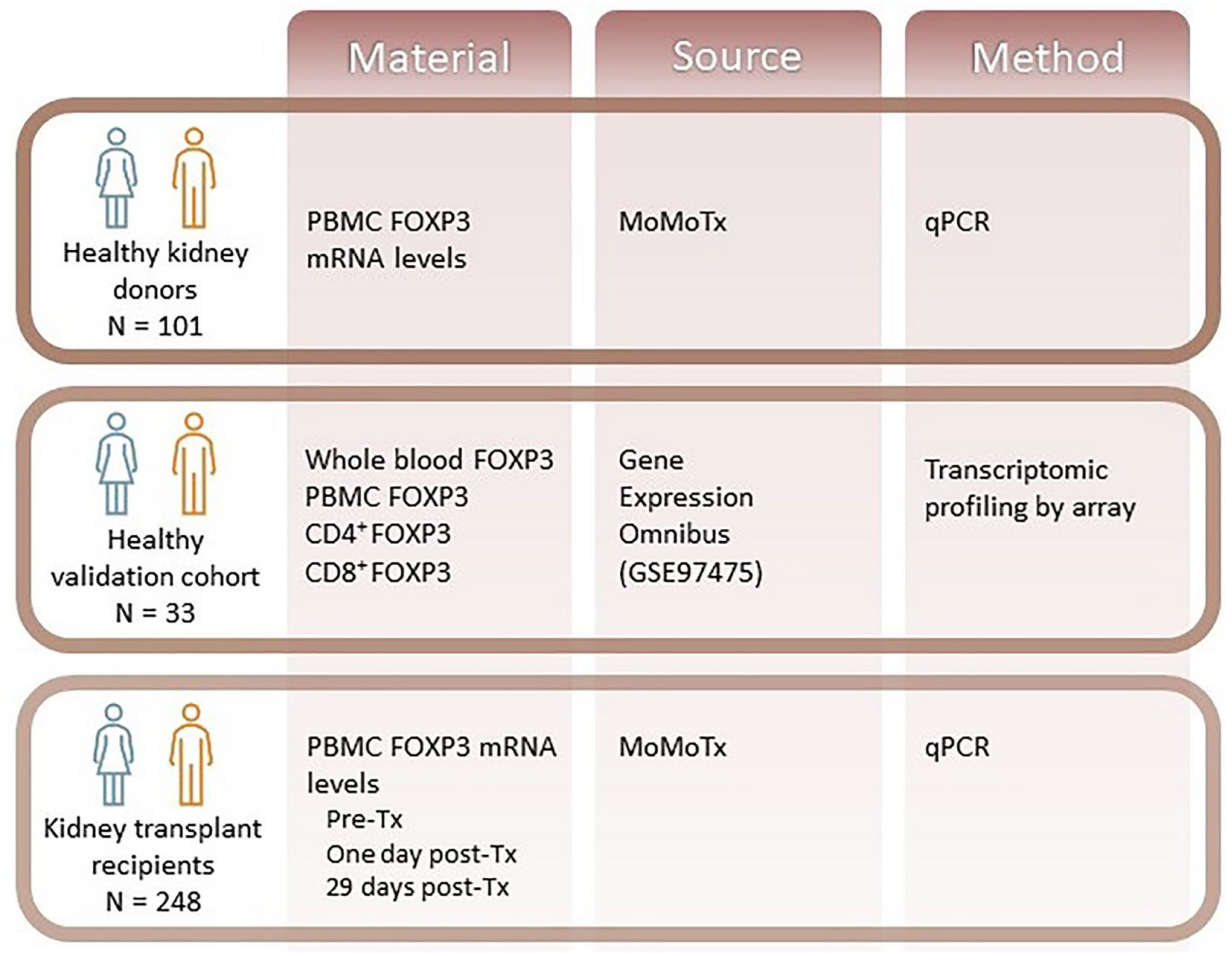
Overview of cohorts used for the analysis and of acquired materials, the source of the material and the methods used to measure FOXP3 in each cohort. FOXP3: Forkhead box P3. PBMC: peripheral blood mononuclear cells. MoMoTx: the molecular monitoring after kidney transplantation project. qPCR: quantitative polymerase chain reaction.

Of the 617 MoMoTx KTRs enrolled from January 2011 until August 10. 2021, 248 had available blood samples pre-transplant, one day after transplant and 29 days after transplant, and were included in the final analysis. Of the 248 KTRs included in the analysis, 81 (32%) were females and 167 (68%) were males. Demographic, anthropometric, and clinical data are detailed in Table 1. Compared to male KTRs, female KTRs had lower admission weight (72 kg [62 to 85] vs 85 kg [75 to 96], p < 0.01), height (165 cm [162 to 170] vs 180 [175 to 185], p < 0.01), pre-transplant serum creatinine (573 µmol/L [476 to 744] vs 776 µmol/L [633 to 988], p < 0.01) and serum creatinine the first day post-transplant (310 µmol/L [209 to 478] vs 445 [340 to 616], p < 0.01). The difference between pre-transplant serum creatinine and serum creatinine measured the first day post-transplant was similar in female and male KTRs (p > 0.90). Remaining clinical characteristics were also similar in female and male KTRS, including distributions of age (p > 0.90), underlying kidney disease (p > 0.90), prior kidney transplantation (p > 0.90), donor type (p > 0.90), diabetic comorbidity (p > 0.90), hypertension (p > 0.90), vascular atherosclerotic disease (p > 0.90), induction and maintenance therapies (Fig. 1).

**Table 1:** Baseline characteristics of kidney transplant recipients, compared according to sex.

Variable	All to n = 248	Female to N = 81	Male to N = 167	P-value	Adjusted p-value
Age (years)	51 [41 to 62]	53 [43 to 62]	50 [40 to 62]	0.50	> 0.90
Weight (kg)	81 [71 to 93]	72 [62 to 85]	85 [75 to 96]	< 0.01	< 0.01
Height (kg)	175 [168 to 183]	165 [162 to 170]	180 [175 to 185]	< 0.01	< 0.01
Prior kidney transplantation, N (%)	37 (15%)	14 (17%)	23 (14%)	0.50	> 0.90
Underlying kidney disease, N (%)				0.15	> 0.90
Glomerulonephritis or vasculitis	85 (34%)	31 (38%)	54 (32%)		
Diabetic nephropathy	34 (14%)	6 (7.4%)	28 (17%)		
Hypertensive nephropathy	30 (12%)	7 (8.6%)	23 (14%)		
Hydronephrosis	14 (5.6%)	5 (6.2%)	9 (5.4%)		
Cystic kidney disease	39 (16%)	18 (22%)	21 (13%)		
Cancer	4 (1.6%)	2 (2.5%)	2 (1.2%)		
Unknown	42 (17%)	12 (15%)	30 (18%)		
Hypertension, N (%)	205 (83%)	60 (74%)	145 (87%)	0.01	> 0.90
Diabetes, N (%)	43 (17%)	9 (11%)	34 (20%)	0.07	> 0.90
Vascular atherosclerotic disease, N (%)	31 (12%)	10 (12%)	21 (13%)	> 0.90	> 0.90
Dialysis type, N (%)				0.50	> 0.90
Pre-emptive	47 (19%)	17 (21%)	30 (18%)		
Hemodialysis	138 (56%)	47 (58%)	91 (54%)		
Peritoneal dialysis	63 (25%)	17 (21%)	46 (28%)		
Dialysis vintage (months)	11 [2 to 24]	12 [3 to 6]	10 [2 to 21]	0.20	> 0.90
Donor type				0.90	> 0.90
Deceased	108 (44%)	36 (45%)	72 (43%)		
Living ABO-compatible	105 (42%)	35 (43%)	70 (42%)		
Living ABO-incompatible	35 (14%)	10 (12%)	25 (15%)		
Donor age (years)	53 [44 to 62] toN_avaliable_ = 166	52 [39 to 64] to N_avaliable_ = 52	54 [46 to 61] to N_avaliable_ = 114	0.30	> 0.90
Donor female sex, N (%)	102 (60%) to N_avaliable_ = 171	26 (49%) to N_avaliable_ = 53	76 (64%) to N_avaliable_ = 118	0.06	> 0.90
HLA total mismatch	3 [2 to 4]	3 [2 to 4]	3 [2 to 5]	0.80	> 0.90
Cold ischemic time (minutes)	750 [545 to 990] to N_avaliable_ = 99	870 [638 to 1020] to N_avaliable_ = 36	720 [540 to 960] to N_avaliable_ = 63	0.20	> 0.90
Pre-transplant serum creatinine to (µmol/L)	730 [547 to 918]	573 [476 to 744]	776 [633 to 988]	< 0.01	< 0.01
Serum creatinine first day post-transplant (µmol/L)	410 [284 to 595]	310 [209 to 478]	445 [340 to 616]	< 0.01	< 0.01
Difference in serum creatinine between pre- and first day post-transplant (µmol/L)	294 [141 to 435]	271 [118 to 375]	314 [148 to 458]	0.11	> 0.90
Immunosuppressive induction therapy, N (%)					
Basiliximab	206 (83%)	65 (80%)	141 (84%)	0.40	> 0.90
Thymoglobulin	37 (15%)	15 (19%)	22 (13%)	0.30	> 0.90
Corticosteroids	23 (9.3%)	27 (33%)	47 (28%)	0.40	> 0.90
Rituximab	52 (21%)	19 (23%)	33 (20%)	0.50	> 0.90
Immunosuppressive maintenance therapy, N (%)					
Tacrolimus	248 (100%)	81 (100%)	167 (100%)		
Myophenalate acid	248 (100%)	81 (100%)	167 (100%)		
Delayed graft function, N (%)	27 (11%)	9 (11%)	18 (11%)	> 0.90	> 0.90
Rejection within the first year post-transplant, N (%)	34 (14%)	13 (16%)	21 (13%)	0.50	> 0.90

Data are presented as median [interquartile range] or number (percent) and compared with Wilcoxon rank sum test, Pearson’s Chi-squared test or Fisher’s exact test were appropriate.

### FOXP3 mRNA expression in females and males

**Figure 2:**
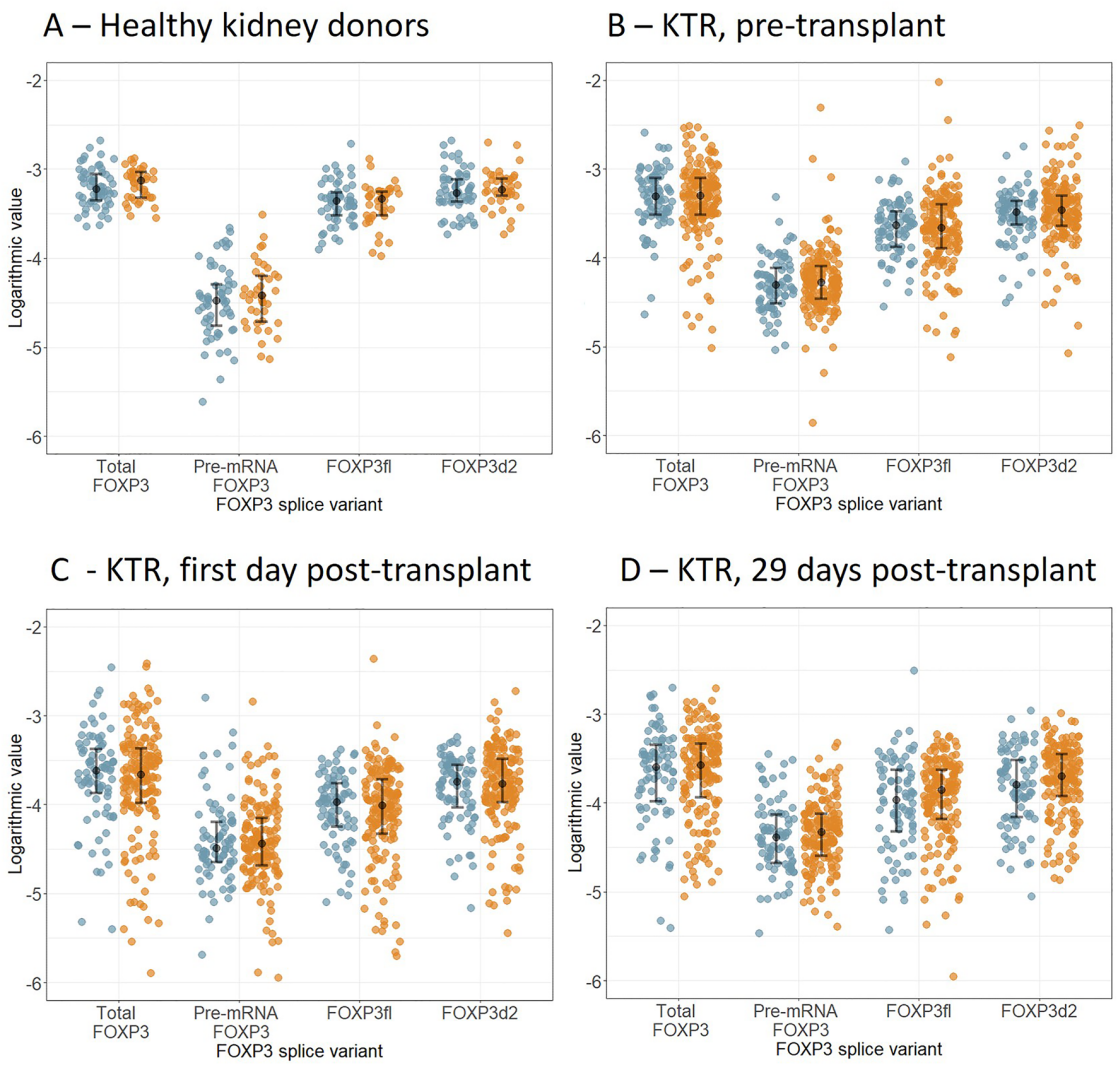
Forkhead box P3 transcript levels in female and male (**A**) healthy kidney donor, (**B**) pre-transplant (Tx) samples of kidney transplant recipients, (**C**) first day post-transplant samples of kidney transplant recipients, and (**D**) 29 days post-transplant samples of kidney transplant recipients.

The expression levels of FOXP3 mRNA were similar in healthy female and male kidney donors (Fig. 2A, Table 2). These findings were homogenous across all FOXP3 variants and were similar for total FOXP3 (p > 0.90), pre-mRNA FOXP3 (p > 0.90), FOXP3fl (p > 0.90), and FOXP3d2 (p > 0.90). In the healthy validation cohort, FOXP3 mRNA levels were measured by array in whole blood, peripheral blood mononuclear cells, CD4^+^ cells and CD8^+^ cells. Here, FOXP3 mRNA levels were also similar in females and males regardless of sample type (Supplementary Table 1). In KTRs, we also found similar distributions of FOXP3 variants in females and males regardless of different sample collection time-points (pre-transplant, first day post-transplant and 29 days post-transplant) (Fig. 2B–D, Table 3).

**Table 2:** Age and logarithmic values of normalized forkhead box P3 (FOXP3) splice variant levels in healthy kidney donors according to sex.

Variable	All (N = 101)	Females (N = 60)	Males (N = 41)	P-value	Adjusted p-value
Age	51 [41 to 59]	51 [42 to 59]	47 [40 to 59]	0.30	> 0.90
Logarithmic value of FOXP3 splice variant					
Total FOXP3	− 3.20 [− 3.32 to − 3.05]	− 3.23 [-3.35 to − 3.06]	− 3.13 [− 3.32 to − 3.03]	0.40	> 0.90
Pre-mRNA FOXP3	− 4.48 [− 4.73 to − 4.24]	− 4.50 [-4.81 to − 4.30]	− 4.42 [− 4.72 to − 4.21]	0.40	> 0.90
FOXP3fl	− 3.35 [− 3.52 to − 3.26]	− 3.36 [-3.52 to − 3.27]	− 3.33 [− 3.52 to − 3.26]	> 0.90	> 0.90
FOXP3d2	− 3.25 [− 3.36 to − 3.11]	− 3.27 [-3.37 to − 3.12]	− 3.23 [− 3.30 to − 3.11]	0.30	> 0.90

Data are presented as median [interquartile range]. Comparisons were performed with Wilcoxon rank sum test. Total FOXP3: detects the two most abundant splice variants, FOXP3fl and FOXP3d2. Pre-mRNA FOXP3: detects pre-mRNA that contain introns. FOXP3fl: detects mature FOXP3 mRNA that includes all exons. FOXP3d2: detects mature FOXP3 mRNA that skip exon 2.

**Table 3:** Forkhead box P3 (FOXP3) splice variant levels in female and male kidney transplant recipients.

Variable	All (N = 248)	Females (N = 81)	Males (N = 167)	p-value	Adjusted p-value
Pre-Tx					
Total FOXP3	− 3.31 [− 3.52 to − 3.10]	− 3.31 [− 3.52 to − 3.11]	− 3.30 [− 3.52 to − 3.10]	> 0.90	> 0.90
Pre-mRNA FOXP3	− 4.29 [− 4.49 to − 4.10]	− 4.32 [− 4.52 to − 4.13]	− 4.27 [− 4.47 to − 4.09]	0.30	> 0.90
FOXP3fl	− 3.66 [− 3.88 to − 3.41]	− 3.64 [− 3.88 to − 3.48]	− 3.67 [− 3.90 to − 3.40]	> 0.90	> 0.90
FOP3d2	− 3.47 [− 3.63 to − 3.32]	− 3.48 [− 3.63 to − 3.36]	− 3.47 [− 3.65 to − 3.30]	0.20	> 0.90
First day post-TX					
Total FOXP3	− 3.65 [− 3.96 to − 3.37]	− 3.62 [− 3.87 to − 3.37]	− 3.66 [− 3.98 to − 3.37]	0.70	> 0.90
Pre-mRNA FOXP3	− 4.46 [− 4.68 to − 4.18]	− 4.50 [− 4.66 to − 4.20]	− 4.45 [− 4.69 to − 4.16]	0.80	> 0.90
FOXP3fl	-4.00 [-4.33 to -3.73]	-3.97 [-4.25 to -3.76]	− 4.01 [− 4.34 to − 3.72]	0.70	> 0.90
FOP3d2	− 3.75 [− 3.98 to − 3.52]	− 3.74 [− 4.03 to − 3.55]	− 3.77 [− 3.97 to − 3.49]	0.60	> 0.90
29 days post-TX					
Total FOXP3	− 3.58 [− 3.97 to − 3.33]	− 3.60 [− 3.98 to − 3.35]	− 3.53 [− 3.94 to − 3.33]	0.90	> 0.90
Pre-mRNA FOXP3	− 4.36 [− 4.62 to − 4.13]	− 4.40 [-4.69 to − 4.13]	-4.33 [-4.60 to − 4.13]	0.20	> 0.90
FOXP3fl	− 3.89 [− 4.24 to − 3.63]	-3.96 [-4.32 to -3.63]	− 3.85 [-4.18 to − 3.63]	0.20	> 0.90
FOP3d2	− 3.72 [− 3.97 to − 3.45]	− 3.80 [− 4.16 to − 3.51]	− 3.70 [− 3.93 to − 3.45]	0.09	> 0.90

Data are presented as median [interquartile range]. Comparisons were performed with Wilcoxon rank sum test and adjusted for multiple testing using Bonferroni’s correction approach. Total FOXP3: detects the two most abundant splice variants, FOXP3fl and FOXP3d2. Pre-mRNA FOXP3: detects pre-mRNA that contain introns. FOXP3fl: detects mature FOXP3 mRNA that includes all exons. FOXP3d2: detects mature FOXP3 mRNA that skip exon 2.

### FOXP3 mRNA expression in females and males according to age-subgroups

To explore the potential effects of age on FOXP3 expression in females and males, we performed sub-group analysis according to age groups. Study participants were subdivided into those aged 45 years or older, and those aged 44 years or younger. In healthy kidney donors, the subdivision of females and males into age-groups did not alter previous results, as FOXP3 mRNA levels were similar in females and males in each age-group (Supplementary Fig. 1A, Supplementary Table 2). In the healthy validation cohort (Supplementary Table 3), FOXP3 mRNA levels were also similar in females and males in all cell types and in both age-groups. In KTRs, the distributions of FOXP3 variants were also similar in pre-transplant samples, day one post-transplant samples, and day 29 post-transplant samples regardless of sex and age-group (Supplementary Fig. 1 B-D, Supplementary Table 4).

We also compared females aged 44 years or younger with females aged 45 years or older, and males aged 44 years or younger with males aged 45 years or older in healthy kidney donors. The FOXP3 mRNA levels were also similar despite of differing age-groups (Supplementary Table 5), differing cell types and age-groups in the healthy validation cohort (Supplementary Table 6), and differing time-points of sample collection and age-groups in KTRs (Supplementary Table 7).

### Fold difference analysis of FOXP3 splice variants in KTRs compared to healthy controls

To explore differences in FOXP3 expression in KTRs compared to healthy controls, we performed 2^−ΔΔCq^ calculations. In this analysis, the calculated number describes the fold difference between KTR FOXP3 expression compared to the mean FOXP3 expression in healthy controls. By design, the mean FOXP3 expression of healthy controls is about one. Thus, 2^-ΔΔCq^ values above one indicates increased expression, or higher values compared to healthy controls, and values below one indicates decreased expression or lower values compared to healthy controls.

The fold difference analysis revealed that pre-transplant samples of KTRs had lower FOXP3fl (0.48 [ IQR 0.25 to 0.90]) and lower FOXP3d2 (0.54 [IQR 0.38 to 0.80]) expression compared to healthy controls (Supplementary Fig. 2). The median fold difference in total FOXP3 expression in KTRs was 0.76 [IQR 0.48 to 1.22]. FOXP3fl, FOXP3d2 and total FOXP3 expression declined at the first day post-transplant and increased thereafter at the 29^th^ day post-transplant (Table 4). In agreement with prior analysis, the fold difference of FOXP3 expression was similar in females and males.

**Table 4:** Comparison of fold difference of female and male KTR FOXP3 mRNA levels relative to sex-matched mean FOXP3 mRNA levels of healthy kidney donors.

Variable	All (N = 248)	Females (N = 81)	Males (N = 167)	P-value	Adjusted p-value
Pre-transplant					
Total FOXP3	0.76 [0.48 to 1.22]	0.79 [0.50 to 1.27]	0.75 [0.47 to 1.21]	0.70	> 0.90
Pre-mRNA FOXP3	2.02 [1.26 to 3.11]	2.26 [1.44 to 3.59]	1.93 [1.24 to 2.90]	0.07	0.90
FOXP3fl	0.48 [0.25 to 0.90]	0.51 [0.26 to 0.70]	0.47 [0.24 to 0.91]	> 0.90	> 0.90
FOXP3d2	0.54 [0.38 to 0.80]	0.54 [0.42 to 0.77]	0.55 [0.37 to 0.82]	> 0.90	> 0.90
First day post-transplant					
Total FOXP3	0.35 [0.17 to 0.66]	0.37 [0.22 to 0.67]	0.33 [0.16 to 0.65]	0.50	> 0.90
Pre-mRNA FOXP3	1.4 [0.8 to 2.8]	1.5 [1.0 to 3.0]	1.3 [0.7 to 2.5]	0.06	0.80
FOXP3fl	0.21 [0.09 to 0.43]	0.24 [0.11 to 0.38]	0.20 [0.09 to 0.44]	0.80	> 0.90
FOXP3d2	0.29 [0.16 to 0.51]	0.32 [0.15 to 0.51]	0.28 [0.17 to 0.51]	0.90	> 0.90
29 days post-transplant					
Total FOXP3	0.41 [0.17 to 0.71]	0.44 [0.17 to 0.70]	0.40 [0.18 to 0.72]	0.70	> 0.90
Pre-mRNA FOXP3	1.78 [0.92 to 2.88]	1.88 [0.97 to 3.57]	1.73 [0.91 to 2.78]	0.40	> 0.90
FOXP3fl	0.28 [0.12 to 0.55]	0.22 [0.08 to 0.55]	0.32 [0.13 to 0.55]	0.20	> 0.90
FOXP3d2	0.31 [0.16 to 0.56]	0.28 [0.12 to 0.53]	0.33 [0.18 to 0.58]	0.30	> 0.90

Data are presented as median [interquartile range]. Comparisons were performed with Wilcoxon rank sum test and adjusted for multiple testing using Bonferroni’s correction approach. Total FOXP3: detects the two most abundant splice variants, FOXP3fl and FOXP3d2. Pre-mRNA FOXP3: detects pre-mRNA that contain introns. FOXP3fl: detects mature FOXP3 mRNA that includes all exons. FOXP3d2: detects mature FOXP3 mRNA that skip exon 2.

**Figure 3:**
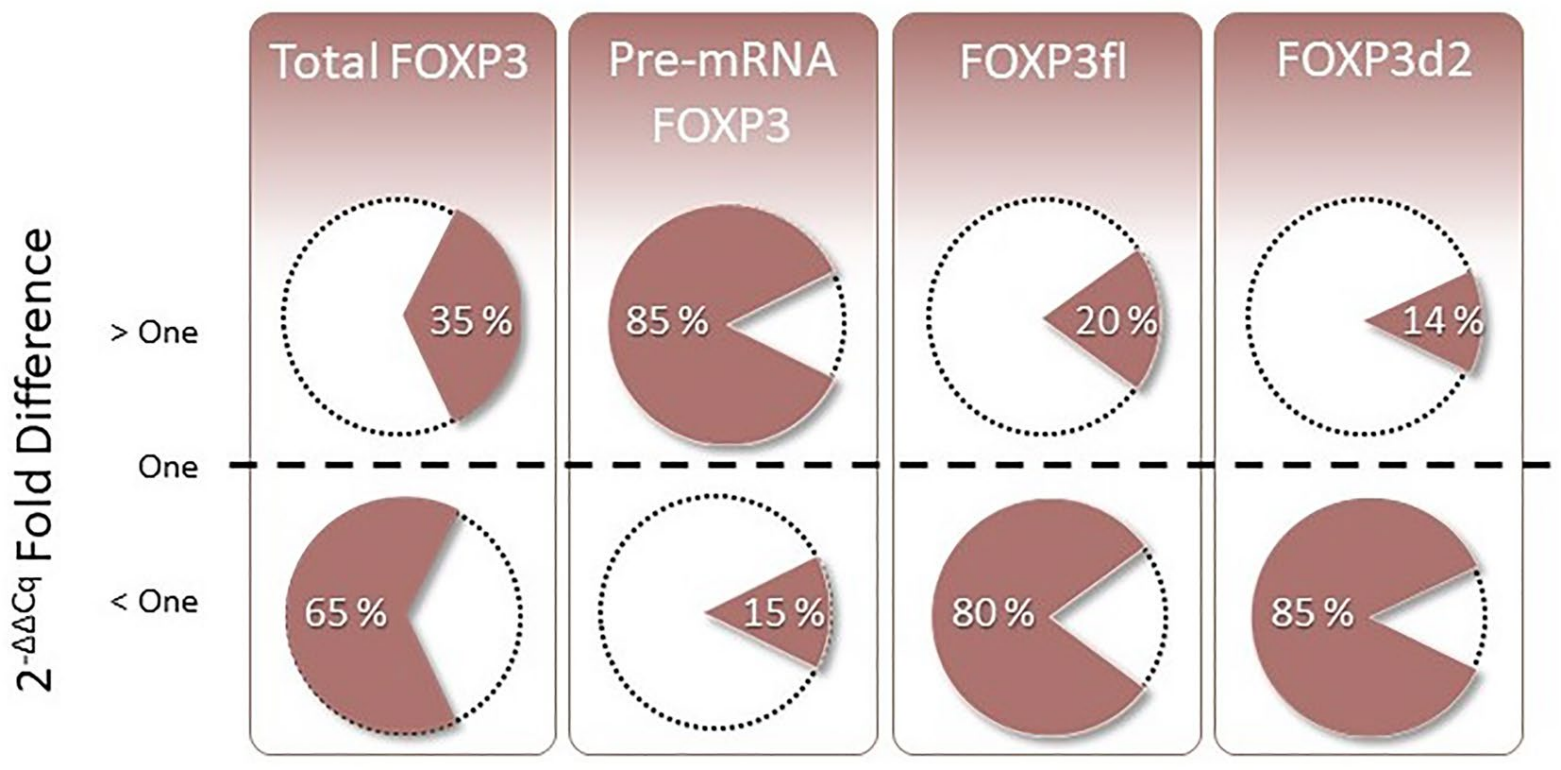
Percentage of kidney transplant recipients according to fold difference of FOXP3 transcript variant expression relative to healthy kidney donor mean FOXP3 expression (dashed line).

In contrast to the lower expression of spliced FOXP3 (FOXP3d2, FOXP3fl and total FOXP3) in KTRs compared to healthy controls, pre-mRNA FOXP3 expression was higher in KTRs pre-transplant samples (2.02 [IQR 1.26 to 3.11]). This contrast can be appreciated by considering the number of KTRs with FOXP3 fold differences above or below one (Fig. 3, Table 5). The higher values of FOXP3 pre-mRNA in KTRs pre-transplant are also evident when comparing the logarithmic values of normalized pre-mRNA FOXP3 of KTRs and healthy controls (Supplementary Table 8).

**Table 5:** Number and percent of transplant recipients with FOXP3 2^-ΔΔCt^ fold difference > 1 according to timepoint of sample collection and FOXP3 variant.

FOXP3 variant	Pre-transplant	First day post-transplant	29 days post-transplant
Total FOXP3	88 (35.48%)	33 (13.31%)	38 (15.32%)
Pre-mRNA FOXP3	212 (85.48%)	162 (65.32%)	180 (72.58%)
FOXP3fl	50 (20.16%)	8 (3.23%)	15 (6.05%)
FOXP32	35 (14.11%)	9 (3.63%)	13 (5.24%)

Fold difference was calculated with 2^−ΔΔCt^. Female and male KTR FOXP3 mRNA levels were calculated relative to sex-matched mean FOXP3 mRNA levels of healthy kidney donors. Data are presented as median [interquartile range]. Comparisons were performed with Wilcoxon rank sum test and adjusted for multiple testing using Bonferroni’s correction approach. Total FOXP3: detects the two most abundant splice variants, FOXP3fl and FOXP3d2. Pre-mRNA FOXP3: detects pre-mRNA that contain introns. FOXP3fl: detects mature FOXP3 mRNA that includes all exons. FOXP3d2: detects mature FOXP3 mRNA that skip exon 2.

Like total FOXP3, FOXP3fl and FOXP3d2 levels, the fold difference of pre-mRNA FOXP3 decreased the first day post-transplant 1.4 [IQR 0.8 to 2.8], from which it increased slightly the 29^th^ day post-transplant (1.78 [IQR 0.92 to 2.88]).

## Discussion

The FOXP3 transcription factor is essential for T regulatory cell differentiation, phenotype stability, and suppressive function. In turn, T regulatory cells are crucial for immunologic tolerance of auto- and alloantigens. To examine a potential association between biological sex and FOXP3 expression, we conducted a sex-based descriptive analysis in two healthy populations and in one population of KTRs. Our data shows that FOXP3 mRNA levels were similar in biological females and males, regardless of time-point of measurement relative to kidney transplantation in KTRs, different splice variants, or age-groups. However, our data showed that KTRs express higher levels of pre-mRNA FOXP3 and lower levels of mature mRNA FOXP3 (FOXP3fl and FOXP3d2) compared to healthy donors.

To the best of our knowledge, our investigation is the first to examine FOXP3 splice variants in healthy participants and in KTRs. Two recent publications have reported conflicting results regarding FOXP3 mRNA levels in healthy females and males. In support of our data Robinson et. al. report no difference in FOXP3 mRNA levels between healthy biological females and males^16^. In contrast, Singh et. al. reported that FOXP3 mRNA levels were 2–3 folds higher in healthy biological males compared to age-matched healthy females^15^. These conflicting results may be explained by methodological differences. Robinson et. al. measured FOXP3 mRNA levels in FACS-sorted T regulatory cells (CD4^+^CD25^+^CD127^low^) from five biological females and five biological males using RNA sequencing^16^. Singh et. al. measured FOXP3 mRNA levels using real time polymerase chain reaction on RNA extracted from FACS-sorted T regulatory cells (CD4^+^CD25^+hi^) from eight healthy males and 10 age-matched healthy females ^15^. The difference in gating of FACS-sorting of T regulatory cells, may have contributed to the differing results. We measured FOXP3 mRNA levels in a larger cohort of healthy individuals that consisted of 60 females and 41 males, using quantitative polymerase chain reaction in peripheral blood mononuclear cells. In peripheral blood, most FOXP3 expression is limited to T regulatory cells^17,18^, which can be appreciated by a tau score of 0.94 (www.proteinatlas.org/ENSG00000049768-FOXP3/immune+cell). The level of FOXP3 expression is thus considered a reliable marker of regulatory T cell abundance ^17^, and is furthermore correlated with regulatory T cell function^19^. Moreover, we validated our findings in a validation cohort, where FOXP3 was measured by array in specific cell subsets. The healthy validation cohort consisted of 20 females and 13 males. Here too, FOXP3 mRNA levels were similar in healthy females and males regardless of cell type. Thus, our analysis contributes two larger cohorts which show that FOXP3 mRNA is expressed at similar levels in healthy females and males.

FOXP3 expression seems to increase in biological females of reproductive age with increasing estrogen levels^15,16,20,21^. Measurements of estrogen levels in study participants might have been beneficial but was out of the scope of this article. However, we observed no differences in FOXP3 mRNA splice variant expression in older and younger females. As sex hormone levels decrease with age^22^, and as estrogen levels stabilize at lower levels in older females^23^, we expected to see a decline in FOXP3 mRNA levels in older healthy females, but this was not the case. The comparison of FOXP3 expression in older and younger males did not reveal any differences either. This may imply that physiological levels of estrogen and sex hormones affect FOXP3 mRNA levels to a lesser extent. Moreover, end stage kidney disease leads to unfluctuating estrogen levels in females of reproductive age, although their estrogen levels are comparable to those of a healthy age-matched female in the follicular phase of the menstrual cycle^24^. We measured FOXP3 levels in KTRs pre- and post-transplant. In this group also, FOXP3 splice variant levels were similar in females across age-groups. Thus, in a population of females prone to unfluctuating estrogen levels, FOXP3 splice variant levels did not differ in different age-groups. This finding may also suggest that the effect of estrogen plays a small role in regulation of levels on FOXP3 mRNA in biological females.

Immunosuppressive medicines decrease FOXP3 levels either directly or indirectly by depletion of T regulatory cells^12–14^. Measurements of FOXP3 splice variant levels in KTRs revealed similar expression levels in females and males pre- and post-transplant. FOXP3 splice variant levels were also similar in sub-group analysis across age-groups. As treatment with immunosuppressive medicines are standardized in KTRs, our results suggest that the effects of immunosuppressive medicines on FOXP3 splice variant fluctuations do not vary according to biological sex or age.

In comparison of KTR FOXP3 splice variant fold difference compared to sex-matched healthy controls, we observed that KTRs had higher pre-transplant pre-mRNA FOXP3 levels, and lower pre-transplant mature FOXP3 mRNA levels (total FOXP3, FOXP3fl, FOXP3d2). Mature mRNA is processed from pre-mRNA by the spliceosome, a large ribonucleic protein complex consisting of more than 100 individual components^25,26^. The assembly, efficiency, and regulation of the spliceosome is highly complex and is regulated by epigenetic, intrinsic and extrinsic factors^26^. Furthermore, the actions of the spliceosome can be influenced by environmental factors in eukaryotic cells^27^. Pre-transplant samples of KTRs represent individuals afflicted by renal dysfunction and end stage renal disease, which implies higher risks of uremia, metabolic acidosis, and hormonal changes^28^. The consequences of renal dysfunction have been shown to alter the transcriptome^29^ and the blood proteome^30,31^. Thus, the consequences of end stage renal disease may have altered FOXP3 pre-mRNA and mature mRNA levels. The observed higher levels of pre-mRNA FOXP3 levels and lower mature FOXP3 levels in our data may suggest higher pre-mRNA synthesis, lower pre-mRNA degradation, lower spliceosome efficiency or higher degradation of mature FOXP3 mRNA. The difference in pre-mRNA and mature mRNA levels are consequential because protein levels, and consequently cell functions, are correlated with mature mRNA levels in steady-state^32^. Therefore, uncovering the underlying mechanisms may unearth therapeutic targets or insight into the pathogenesis of diseases related to kidney dysfunction.

Our study has limitations that must be considered. Firstly, although the sex-based analysis was conducted in a large healthy cohort, a large KTR cohort, and a healthy validation cohort, the sub-group analysis according to age reduces statistical power. Thus, if increasing age has a limited effect on FOXP3 mRNA splice variant expression, it may need a large study population to be appreciated. Second, replication of our results is warranted in non-European populations and may therefore not be generalized. Third, although mRNA levels correlate with protein levels in the steady state^32^, there may be sex-based differences in FOXP3 splice variant functions. Forth, we assessed FOXP3 at early stages after kidney transplantation in KTRs, and FOXP3 mRNA levels may be different at later stages after kidney transplantation. Finally, flow cytometry data would have provided further information regarding the functionality of regulatory T cells in healthy participants. Sex-based analysis of FOXP3 mRNA splice variant levels revealed similar levels of FOXP3 expression in males and females in two healthy cohorts and a cohort of kidney transplant recipients. The similarity in FOXP3 levels did not seem to be affected by age, although a larger cohort may reveal a smaller impact of age on FOXP3 expression. Analysis of FOXP3 splice variant levels suggest that the effects of immunosuppressive medical regiments are similar in females and males, as FOXP3 splice variant levels remained similar between the sexes in post-transplant samples. Lastly, although kidney transplant recipients expressed higher pre-mRNA FOXP3 levels compared to healthy sex-matched controls, they expressed lower levels of mature FOXP3 mRNA. This may suggest higher pre-mRNA synthesis, lower pre-mRNA degradation, lower spliceosome efficiency or higher degradation of mature FOXP3 mRNA.

## Methods

### Material and ethical approval

We used several cohorts for the analysis (Fig. 1). To analyze FOXP3 expression in healthy individuals, we used blood samples from healthy kidney donors enrolled in the molecular monitoring after kidney transplantation project (MoMoTx)^33–35^. To validate our findings in healthy subjects (validation cohort), we used a publicly available dataset (GSE97475). Information on GSE97475 was obtained from the gene expression omnibus (ncbi.nlm.nih.gov/geo/) and accessed via the R package GEOquery June 10. 2023^36^. Finally, to analyze FOXP3 expression in KTRs, we used blood samples collected from KTRs enrolled in MoMoTx.

Details describing MoMoTx have been published previously^33–35,37^. MoMoTx is an ongoing project that prospectively enrolls incident KTRs and healthy kidney transplant donors. All participants are enrolled at Odense university hospital, Denmark. KTRs and healthy donors are considered eligible for inclusion if they are older than 18 years, and if they can provide informed consent. Re-transplantations are considered as new study participants, and multiple organ transplants are excluded. Blood samples are collected from all participants. From healthy kidney donor participants, a blood sample is collected before kidney donation, while blood samples are collected before and after transplantation from KTRs. Data concerning sex and age are collected from healthy kidney donors, while clinical data is collected from KTRs. The study protocol has been approved by the local ethics committee (Den Videnskabsetiske Komite for Region Syddanmark, Project-ID: 20,100,098) and is in accordance with the declarations of Helsinki and Istanbul. All participants agreed for enrollment with written informed consent.

In this project, we included KTRs enrolled in MoMoTx from January 2011 until August 10. 2021 that had available blood samples drawn pre-transplant, one day post-transplant and 29 days post-transplant. We extracted anthropometric and clinical data including: age (years); biological sex; weight at admission (kg), height at admission (cm), presence of prior kidney transplantation (yes or no); underlying kidney disease (Glomerulonephritis, hypertensive nephropathy, diabetic nephropathy, hydronephrosis, cystic kidney disease, cancer or unknown); comorbidities including hypertension, diabetes and vascular atherosclerotic disease (presence of one or a combination of cardiovascular or cerebrovascular disease, and peripheral arterial disease); Dialysis type (pre-emptive, hemodialysis or peritoneal dialysis); Dialysis duration (months, total); Kidney donation type (deceased, living ABO-compatible or living ABO-incompatible); human leukocyte antigen total mismatch number; pre-transplant serum creatinine level (µmol/L); serum creatinine level the first day post-transplant (µmol/L); delayed graft function (need for dialysis within the first week post-transplant); rejection within the first year post-transplant (presence of the Danish ICD-10 codes DT861, DT861B, DT868 or DT869 within one year post-transplant); immunosuppressive induction therapeutics (basiliximab, thymoglobulin, corticosteroids or rituximab); immunosuppressive maintenance therapy (tacrolimus and mycophenolate acid). In available donor data we extracted donor age (years), biological sex, and cold ischemic time (minutes).

### Measurements of FOXP3 expression in MoMoTx participants

Blood collected from KTRs and healthy kidney donors was collected in heparinized tubes. Using density gradient centrifugation with Histopague (Sigma-Aldrich, St. Louis, MO, USA; density 1.077 g/mL), we isolated peripheral blood mononuclear cells. Peripheral blood mononuclear cells were subsequently washed in Hanks’ balanced salt solution (Thermo Fisher scientific, Waltham, MA, USA), centrifuged and suspended in TRIzol (Invitrogen, Thermo Fisher Scientific, Waltham, MA, USA). We proceeded to extract ribonucleic acid with the RNeasy Mini kit including RNase-free DNase set (Qiagen, Hilden, Germany) according to the manufacturer’s instructions. Complementary DNA was then synthesized with QuantiTect Reverse Transcription kit (Qiagen, Hilden, Germany). Finally, quantitative reverse transcription polymerase chain reaction was performed with LightCycler 96 (Roche Diagnostics, Basel, Switzerland), using SYBR green.

We used four primer pairs in quantitative reverse transcription polymerase chain reaction. We used a primer pair to detect all mature mRNA FOXP3 splice variants (total FOXP3), a primer to detect pre-mature mRNA FOXP3 (pre-mRNA FOXP3), a primer pair to detect mature full length FOXP3 mRNA (FOXP3fl), and a primer pair to detect mature FOXP3 mRNA in which exon 2 was removed in RNA-splicing (FOXP3d2). All primers were normalized to β-actin. The primer pair sequences were as follows: total FOXP3 forward 5’-GTGGCCCGGATGTGAGAAG-3’ and reverse 5’-GGAGCCCTTGTCGGATGATG-3’; Pre-mRNA FOXP3 forward 5’-TTCACCTGTGTATCTCACGCA-3’ and reverse 5’-gacagcggaggaagtagcta-3’; FOXP3fl forward 5’-AAAGCCTCAGACCTGCTG-3’ and reverse 5’-AGGGTGCCACCATGACTA-3’; FOXP3d2 5’-CAGCTGCAGCTCTCAACGGTG-3’ and reverse 5’-GCCTTGAGGGAGAAGACC-3’; β-actin forward 5’-GGACTTCGAGCAAGAGATGG-3’ and reverse 5’-AGCACTGTGTTGGCGTACAG-3’. The reverse transcription settings were as follows: pre-incubation temperature was 95°C and was set for 10 min, 55 cycles were performed with denaturation at 95°C for 10 s, annealing temperature at 63°C for 10 s and extension at 72°C for 10 s. Each sample was measured in duplicate wells. The results were analyzed with LightCycler 96 Software 1.1 (Roche Diagnostics, Denmark). Reactions with multiple melting peaks or irregular melting peaks compared to control reactions were repeated and removed from further analyses if persistent. Finally, we calculated normalized target FOXP3 levels relative to β-actin using the equation:

Normalized ratio = ET^(CqR-CqT)^.

ET, efficiency of target amplification; CqT and CqR, mean quantification cycle at target/reference detection.

Normalized FOXP3 levels were converted to a logarithmic scale to ease interpretation of results.

### Statistical analysis

Categorical data are presented as number (percent) and compared with Fisher’s exact test or Chi-squared test where appropriate. Continuous variables were assessed for normality with QQ-plots, presented as median [interquartile range (IQR)], and tested with Wilcoxon’s rank-sum test.

In subgroup analysis, we analyzed FOXP3 mRNA level expression according to age-subgroups. Study participants were dichotomized into those who were 44 years old or younger at time of inclusion, and those who were 45 years or older at time of inclusion. We chose the cut-off of 45 based on the literature^2^, and because endocrine changes characteristic of perimenopause begin at approximately age 45 in females^38,39^. Statistical analyses were adjusted for multiple testing using Bonferroni correction. All statistical analyses were performed in R (version 4.2.3, R Foundation for Statistical Computing, Vienna, Austria).

To describe the fold difference of FOXP3 mRNA level expression in KTRs compared to healthy donor FOXP3 mRNA levels, we used the 2^−ΔΔCq^ method^40^. The formulae used to calculate ΔΔCq were as follows:

$$ \Delta \Delta {\text{Cq}}{\mkern 1mu} \,{ = }{\mkern 1mu} {\text{(Cq}}_{{{\text{FOXP3}}}} \, - \,{\text{Cq}}_{{\beta {\text{ - actin}}}} )_{{\text{kidney transplant recipient}}} \, - \,{\text{mean}}\,\Delta {\text{Cq}}_{{\text{healthy control}}} $$

$$ {\text{mean }}\Delta {\text{Cq}}_{{\text{healthy control}}} {\mkern 1mu} { = }\,{\text{arithmetic mean of (Cq}}_{{{\text{FOXP3}}}} { - }\,{\text{Cq}}_{{\beta {\text{ - actin}}}} {\text{) in healthy controls}} $$

Cq  mean quantification cycle.

Female KTR Cq values were compared to mean ΔCq of healthy female kidney donors, and male KTR CT values were compared to mean ΔCq of healthy male kidney donors.

### Supplementary Information

Supplementary Information.

## Data Availability

Data is provided within the manuscript and supplementary information files.

## Abbreviations

FOXP3: Forkhead box P3
FOXP3d2: Forkhead box P3 in which exon two has been skipped by RNA-splicing
FOXP3fl: Full length Forkhead box P3 in which all coding exons are expressed
KTR: Kidney transplant recipient
MoMoTx: Molecular monitoring after kidney transplantation project
