# Supplementary material for: FOXP3 splice variant expression in males and females in healthy populations and in kidney transplant recipients
Source: Sci Rep. 2024 May 27;14:12112. doi: 10.1038/s41598-024-62149-1 (PMC11130272; doi:10.1038/s41598-024-62149-1)

Saleh et al. FoxP3 MF Suppl. data

Table of contents

Supplementary table 1 - Forkhead box P3 (FOXP3) mRNA expression in a publicly available dataset (GSE97475) according to participant sex.

Supplementary table 2 – Forkhead box P3 (FOXP3) levels in healthy female and male kidney donors according to age-group.

Supplementary table 3 - Forkhead box P3 (FOXP3) levels in healthy females and males according to age-group in the healthy validation group.

Supplementary table 4 - Forkhead box P3 (FOXP3) splice variant levels in older and younger kidney transplant recipients according to sex.

Supplementary table 5 - Comparison of expression of normalized forkhead box P3 (FOXP3) levels in younger vs older healthy kidney donors.

Supplementary table 6 - Comparison of forkhead box P3 (FOXP3) levels in healthy females and males, from the healthy validation group, according to age-group.

Supplementary table 7 - Comparison of normalized forkhead box P3 (FOXP3) splice variant levels in male and female kidney transplant recipients according to age-group.

Supplementary table 8 - Comparison of forkhead box P3 (FOXP3) expression in pre-transplant samples from kidney transplant recipients and healthy kidney donor controls.

Supplementary Figure 1 - Distrubiton of forkhead box P3 transcript levels according to sex and age-subgroup in females and males.

Supplementary Figure 2 – Scatterplot of fold difference of pre-transplant forkhead box P3 transcript levels in kidney transplant recipients relative to healthy kidney donor levels.

Supplementary Table 1 – Forkhead box P3 (FOXP3) mRNA expression in a publicly available dataset (GSE97475) according to participant sex.

**Cell type**	**All participants (N = 33)**	**Female participants (N = 20)**	**Male participants (N = 13)**	**P-value**	**Adjusted P-value**
Age	26 (19 to 35)	27 (22 to 41)	21 (19 to 29)	0.13	0.65
Logarithmic value of FOXP3 according to cell origin					
Peripheral blood mononuclear cells	4.69 (4.58 to 4.84)	4.73 (4.58 to 4.87)	4.66 (4.60 to 4.77)	0.60	> 0.90
CD4 cells	4.79 (4.56 to 5.03) (unkown = 4)	4.79 (4.57 to 5.08) (unkown = 2)	4.66 (4.55 to 4.91) (unkown = 2)	0.60	> 0.90
CD8 cells	4.80 (4.62 to 4.96) (unkown = 3)	4.72 (4.60 to 5.06) (unkown = 1)	4.85 (4.63 to 4.90) (unkown = 2)	0.90	> 0.90
Whole blood	4.65 (4.55 to 4.81) (unkown = 5)	4.58 (4.58 to 4.83) (unkown = 3)	4.61 (4.51 to 4.68) (unkown = 2)	0.15	0.75

Table footnotes: Age of the participants was similar. We found information concerning the dataset in the gene expression omnibus (ncbi.nlm.nih.gov/geo/). The dataset includes transcriptomic profiling by array of mRNA from peripheral blood of healthy participants (hepatitis B vaccine recipients). According to sample descriptions in the dataset, the platform was Illumina HumanHT-12 V4.0, and data were background subtracted and normalized by quantile normalization with limma's neqc function. The dataset was accessed via R package GEOquery june 10. 2023.

Supplementary Table 2 - Forkhead box P3 (FOXP3) levels in healthy female and male kidney donors according to age-group.

Older (≥ 45 years)				
Variable	Females (N = 42)	Males (N = 24)	p-value	Adjusted p-value
Logarithmic values of				
Total FOXP3	-3.23 [-3.37 to -3.07]	-3.11 [-3.27 to -3.03]	0.20	> 0.90
Pre-mRNA FOXP3	-4.56 [-4.85 to -4.35]	-4.44 [-4.64 to -4.20]	0.20	> 0.90
FOXP3fl	-3.36 [-3.53 to -3.27]	-3.34 [-3.52 to -3.28]	> 0.90	> 0.90
FOP3d2	-3.27 [-3.37 to -3.11]	-3.24 [-3.29 to -3.14]	0.60	> 0.90
Younger (< 45 years)				
	Females (N = 18)	Males (N = 17)	p-value	Adjusted p-value
Logarithmic values of				
Total FOXP3	-3.22 [-3.29 to -3.08]	-3.24 [-3.38 to -3.05]	0.70	> 0.90
Pre-mRNA FOXP3	-4.43 [-4.60 to -4.20]	-4.42 [-4.73 to -4.21]	0.80	> 0.90
FOXP3fl	-3.36 [-3.43 to -3.27]	-3.36 [-3.52 to -3.25]	0.80	> 0.90
FOP3d2	-3.12 [-3.37 to -3.13]	-3.12 [-3.30 to -3.07]	0.30	> 0.90

Table footnotes: Data are presented as median [interquartile range]. Comparisons were performed with Wilcoxon rank sum test and adjusted for multiple testing with Bonferroni's correction. Total FOXP3: detects the two most abundant splice variants, FOXP3fl and FOXP3d2. Pre-mRNA FOXP3: detects pre-mRNA that contain introns. FOXP3fl: detects mature FOXP3 mRNA that includes all exons. FOXP3d2: detects mature FOXP3 mRNA that skip exon 2.

Supplementary Table 3 - Forkhead box P3 (FOXP3) levels in healthy females and males according to age-group in the healthy validation group.

Older (≥ 45 years)				
Variable	Females (N = 5)	Males (N = 2)	p-value	Adjusted p-value
FOXP3 according to cell origin				
Peripheral blood mononuclear cells	4.68 [4.59, 4.94]	4.68 [4.67, 4.68]	> 0.90	> 0.90
CD4 cells	4.80 [4.58, 4.83]	4.88 [4.88, 4.88] (Unknown = 1)	> 0.90	> 0.90
CD8 cells	4.80 [4.57, 4.82]	4.89 [4.89, 4.89] (Unknown = 1)	> 0.90	> 0.90
Whole blood	4.58 [4.50, 4.68] (Unknown = 1)	4.77 [4.72, 4.83]	> 0.90	> 0.90
Younger (< 45 years)				
	Females (N = 15)	Males (N = 11)		p-value
FOXP3 according to cell origin				
Peripheral blood mononuclear cells	4.73 [4.55, 4.84]	4.60 [4.58, 4.83]	0.70	> 0.90
CD4 cells	4.79 [4.56, 5.11] (Unknown = 2)	4.62 [4.55, 4.92] (Unknown = 1)	0.40	> 0.90
CD8 cells	4.71 [4.63, 5.14] (Unknown = 1)	4.83 [4.62, 4.89] (Unknown = 1)	0.30	> 0.90
Whole blood	4.75 [4.63, 4.92] (Unknown = 2)	4.57 [4.50, 4.64] (Unknown = 2)	0.02	0.20

Supplementary Table 4 – Forkhead box P3 (FOXP3) splice variant levels in older and younger kidney transplant recipients according to sex.

Older (≥ 45 years)				
Variable	Females (N = 56)	Males (N = 108)	p-value	Adjusted p-value
Pre-Tx Logarithmic values of				
Total FOXP3				
Pre-mRNA FOXP3	-3.35 [-3.52 to -3.06]	-3.25 [-3.49 to -3.08]	0.50	> 0.90
FOXP3fl	-4.34 [-4.57 to -4.19]	-4.30 [-4.48 to -4.09]	0.20	> 0.90
FOP3d2	-3.66 [-3.89 to -3.47]	-3.65 [-3.94 to -3.38]	0.80	> 0.90
	-3.54 [-3.63 to -3.38]	-3.47 [-3.65 to -3.28]	0.20	> 0.90
First day post-TX logarithmic values of				
Total FOXP3	-3.60 [-3.88 to -3.36]	-3.62 [-3.89 to -3.34]	> 0.90	> 0.90
Pre-mRNA FOXP3	-4.48 [-4.67 to -4.21]	-4.47 [-4.70 to -4.17]	0.90	> 0.90
FOXP3fl	-3.98 [-4.25 to -3.78]	-3.95 [-4.22 to -3.67]	0.50	> 0.90
FOP3d2	-3.75 [-3.97 to -3.54]	-3.70 [-3.93 to -3.47]	0.30	> 0.90
29 days post-TX logarithmic values of				
Total FOXP3	-3.60 [-3.93 to -3.38]	-3.55 [-3.78 to -3.29]	0.50	> 0.90
Pre-mRNA FOXP3	-4.48 [-4.72 to -4.22]	-4.32 [-4.60 to -4.15]	0.07	> 0.90
FOXP3fl	-3.97 [-4.25 to -3.72]	-3.82 [-4.13 to -3.62]	0.08	> 0.90
FOP3d2	-3.83 [-4.15 to -3.56]	-3.68 [-3.88 to -3.45]	0.02	0.30
Younger (< 45 years)				
Variable	Females (N = 25)	Males (N = 59)	p-value	Adjusted p-value
Pre-Tx Logarithmic values of				
Total FOXP3				
Pre-mRNA FOXP3	-3.31 [-3.56 to -3.18]	-3.31 [-3.56 to -3.18]	0.30	> 0.90
FOXP3fl	-4.23 [-4.44 to -4.10]	-4.23 [-4.44 to -4.10]	> 0.90	> 0.90
FOP3d2	-3.69 [-3.85 to -3.40]	-3.69 [-3.85 to -3.40]	0.50	> 0.90
	-3.46 [-3.60 to -3.32]	-3.46 [-3.60 to -3.32]	0.90	> 0.90
First day post-TX logarithmic values of				
Total FOXP3	-3.74 [-4.06 to -3.46]	-3.74 [-4.06 to -3.46]	0.60	> 0.90
Pre-mRNA FOXP3	-4.41 [-4.63 to -4.09]	-4.41 [-4.63 to -4.09]	0.70	> 0.90
FOXP3fl	-4.15 [-4.48 to -3.84]	-4.15 [-4.48 to -3.84]	0.20	> 0.90
FOP3d2	-3.87 [-4.04 to -3.58]	-3.87 [-4.04 to -3.58]	0.60	> 0.90
29 days post-TX logarithmic values of				
Total FOXP3	-3.68 [-4.04 to -3.37]	-3.68 [-4.04 to -3.37]	0.70	> 0.90
Pre-mRNA FOXP3	-4.28 [-4.59 to -4.05]	-4.28 [-4.59 to -4.05]	0.70	> 0.90
FOXP3fl	-3.93 [-4.29 to -3.67]	-3.93 [-4.29 to -3.67]	0.90	> 0.90
FOP3d2	-3.70 [-4.04 to -3.45]	-3.70 [-4.04 to -3.45]	0.70	> 0.90

Table footnotes: Data are presented as median [interquartile range]. Comparisons were performed with Wilcoxon rank sum test. Tx: transplantation. Total FOXP3: detects the two most abundant splice variants, FOXP3fl and FOXP3d2. Pre-mRNA FOXP3: detects pre-mRNA that contain introns. FOXP3fl: detects mature FOXP3 mRNA that includes all exons. FOXP3d2: detects mature FOXP3 mRNA that skip exon 2.

Supplementary Table 5 – comparison of expression of normalized forkhead box P3 (FOXP3) levels in younger vs older healthy kidney donors.

Females				
Variable	Younger (< 45 years, N = 18)	Older (≥ 45 years, N = 42)	p-value	Adjusted p-value
Logarithmic values of				
Total FOXP3	-3.22 [-3.29 to -3.08]	-3.23 [-3.37 to -3.07]	0.70	> 0.90
Pre-mRNA FOXP3	-4.43 [-4.60 to -4.20]	-4.56 [-4.85 to -4.35]	0.20	0.80
FOXP3fl	-3.36 [-3.43 to -3.27]	-3.36 [-3.53 to -3.27]	> 0.90	> 0.90
FOP3d2	-3.26 [-3.37 to -3.13]	-3.27 [-3.37 to -3.11]	0.80	> 0.90
Males				
	Younger (<45 years, N = 17)	Older (≥45 years, N = 24)	p-value	Adjusted p-value
Logarithmic values of				
Total FOXP3	-3.24 [-3.38 to -3.05]	-3.11 [-3.27 to -3.03]	0.40	> 0.90
Pre-mRNA FOXP3	-4.42 [-4.73 to -4.21]	-4.44 [-4.64 to -4.20]	> 0.90	> 0.90
FOXP3fl	-3.31 [-3.52 to -3.25]	-3.34 [-3.52 to -3.28]	0.50	> 0.90
FOP3d2	-3.12 [-3.30 to -3.07]	-3.24 [-3.29 to -3.14]	0.40	> 0.90

Data are presented as median [interquartile range]. Comparisons were performed with Wilcoxon rank sum test. Total FOXP3: detects the two most abundant splice variants, FOXP3fl and FOXP3d2. Pre-mRNA FOXP3: detects pre-mRNA that contain introns. FOXP3fl: detects mature FOXP3 mRNA that includes all exons. FOXP3d2: detects mature FOXP3 mRNA that skip exon 2.

Supplementary Table 6 - comparison of forkhead box P3 (FOXP3) levels in healthy females and males, from the healthy validation group, according to age-group.

Female (N = 20)				
Variable	Younger (< 45 years, N = 15)	Older (≥ 45 years, N = 5)	p-value	Adjusted p-value
FOXP3 according to cell origin				
Peripheral blood mononuclear cells	4.73 [4.55, 4.84]	4.68 [4.59, 4.94]	0.90	> 0.90
CD4 cells	4.79 [4.56, 5.11] (Unknown = 2)	4.80 [4.58, 4.83]	0.60	> 0.90
CD8 cells	4.71 [4.63, 5.14] (Unknown = 1)	4.80 [4.57, 4.82]	0.60	> 0.90
Whole blood	4.75 [4.63, 4.92] (Unknown = 2)	4.58 [4.50, 4.68] (Unknown = 1)	0.13	> 0.90
Male (N = 13)				
	Younger (< 45 years, N = 11)	Older (≥ 45 years, N = 2)	p-value	Adjusted p-value
FOXP3 according to cell origin				
Peripheral blood mononuclear cells	4.60 [4.58, 4.83]	4.68 [4.67, 4.68]	> 0.90	> 0.90
CD4 cells	4.62 [4.55, 4.92] (Unknown = 1)	4.88 [4.88, 4.88] (Unknown = 1)	> 0.90	> 0.90
CD8 cells	4.83 [4.62, 4.89] (Unknown = 1)	4.89 [4.89, 4.89] (Unknown = 1)	0.70	> 0.90
Whole blood	4.57 [4.50, 4.64] (Unknown = 2)	4.77 [4.72, 4.83]	0.15	> 0.90

Supplementary Table 7 – Comparison of normalized forkhead box P3 (FOXP3) splice variant levels in male and female kidney transplant recipients according to age-group.

Females				
Variable	younger (<45 years, N = 25)	older (≥45 years, N = 56)	p-value	Adjusted p-value
Pre-Tx Logarithmic values of				
Total FOXP3	-3.25 [-3.45 to -3.14]	-3.35 [-3.52 to -3.06]	0.90	> 0.90
Pre-mRNA FOXP3	-4.22 [-4.44 to -4.09]	-4.34 [-4.57 to -4.19]	0.15	> 0.90
FOXP3fl	-3.58 [-3.77 to -3.51]	-3.66 [-3.89 to -3.47]	0.50	> 0.90
FOP3d2	-3.44 [-3.57 to -3.28]	-3.54 [-3.63 to -3.38]	0.20	> 0.90
First day post-TX logarithmic values of				
Total FOXP3	-3.70 [-3.84 to -3.42]	-3.60 [-3.88 to -3.36]	0.50	> 0.90
Pre-mRNA FOXP3	-4.54 [-4.65 to -4.18]	-4.48 [-4.67 to -4.21]	0.90	> 0.90
FOXP3fl	-3.90 [-4.41 to -3.76]	-3.98 [-4.25 to -3.78]	0.60	> 0.90
FOP3d2	-3.74 [-4.22 to -3.55]	-3.75 [-3.97 to -3.54]	0.60	> 0.90
29 days post-TX logarithmic values of				
Total FOXP3	-3.49 [-4.27 to -3.29]	-3.60 [-3.93 to -3.38]	> 0.90	> 0.90
Pre-mRNA FOXP3	-4.33 [-4.59 to -3.79]	-4.48 [-4.72 to -4.22]	0.07	> 0.90
FOXP3fl	-3.96 [-4.47 to -3.59]	-3.97 [-4.25 to -3.72]	> 0.90	> 0.90
FOP3d2	-3.70 [-4.19 to -3.35]	-3.83 [-4.15 to -3.56]	0.40	> 0.90
Males				
Variable	Young (<45 years to N = 59)	Older (≥45 years to N = 108)	p-value	Adjusted p-value
Pre-Tx Logarithmic values of				
Total FOXP3	-3.31 [-3.56 to -3.18]	-3.25 [-3.49 to -3.08]	0.11	> 0.90
Pre-mRNA FOXP3	-4.23 [-4.44 to -4.10]	-4.30 [-4.48 to -4.09]	0.30	> 0.90
FOXP3fl	-3.69 [-3.85 to -3.40]	-3.65 [-3.94 to -3.38]	0.60	> 0.90
FOP3d2	-3.46 [-3.60 to -3.32]	-3.47 [-3.65 to -3.28]	0.70	> 0.90
First day post-TX logarithmic values of				
Total FOXP3	-3.74 [-4.06 to -3.46]	-3.62 [-3.89 to -3.34]	0.06	0.70
Pre-mRNA FOXP3	-4.41 [-4.63 to -4.09]	-4.47 [-4.70 to -4.17]	0.40	> 0.90
FOXP3fl	-4.15 [-4.48 to -3.84]	-3.95 [-4.22 to -3.67]	0.02	0.40
FOP3d2	-3.87 [-4.04 to -3.58]	-3.70 [-3.93 to -3.47]	0.02	0.40
29 days post-TX logarithmic values of				
Total FOXP3	-3.68 [-4.04 to -3.37]	-3.55 [-3.78 to -3.29]	0.20	> 0.90
Pre-mRNA FOXP3	-4.28 [-4.59 to -4.05]	-4.32 [-4.60 to -4.15]	0.40	> 0.90
FOXP3fl	-3.93 [-4.29 to -3.67]	-3.82 [-4.13 to -3.62]	0.10	> 0.90
FOP3d2	-3.70 [-4.04 to -3.45]	-3.68 [-3.88 to -3.45]	0.50	> 0.90

Data are presented as median [interquartile range]. Comparisons were performed with Wilcoxon rank sum test. Tx: transplantation. Total FOXP3: detects the two most abundant splice variants, FOXP3fl and FOXP3d2. Pre-mRNA FOXP3: detects pre-mRNA that contain introns. FOXP3fl: detects mature FOXP3 mRNA that includes all exons. FOXP3d2: detects mature FOXP3 mRNA that skip exon 2.

Supplemental Table 8 – comparison of forkhead box P3 (FOXP3) expression in pre-transplant samples from kidney transplant recipients and healthy kidney donor controls.

FOXP3 variant	Pre-transplant levels in kidney transplant recipients (N = 248)	Levels in healthy kidney donors (N = 101)	p-value	Adjusted p-value
Total FOXP3	-3.31 [-3.52 to -3.10]	-3.20 [-3.32 to -3.05]	< 0.01	< 0.01
Pre-mRNA FOXP3	-4.29 [-4.49 to -4.10]	-4.48 [-4.73 to -4.24]	< 0.01	< 0.01
FOXP3fl	-3.66 [-3.88 to -3.41]	-3.35 [-3.52 to -3.26]	< 0.01	< 0.01
FOXP3d2	-3.47 [-3.63 to -3.32]	-3.25 [-3.36 to -3.11]	< 0.01	< 0.01

Pre-mRNA FOXP3 values are normalized to β-actin and converted to a logarithmic scale. Data are presented as median [Interquartile range] and compared with Wilcoxon's rank sum test and adjusted with Bonferroni's correction approach.

Supplementary Figure 1 - Distribution of Forkhead box P3 transcript levels (FOXP3) according to sex and age-subgroup in female and male A) healthy kidney donor, B) pre-transplant samples of kidney transplant recipients (KTRs), C) first day post-transplant samples of KTRs, and D) 29 days post-transplant samples of KTRs.

A – Healthy kidney donors

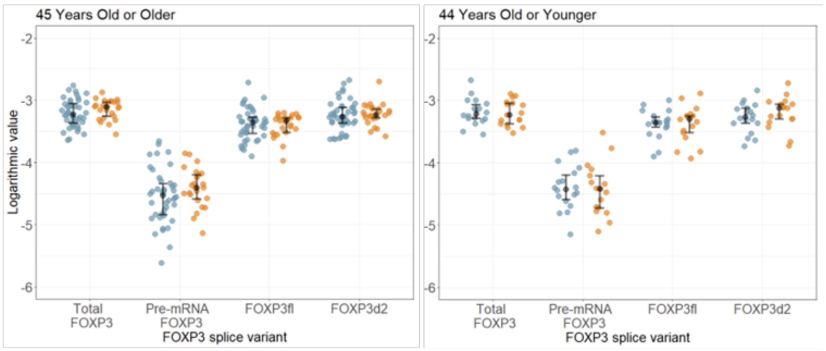

B – KTRs, pre-transplant

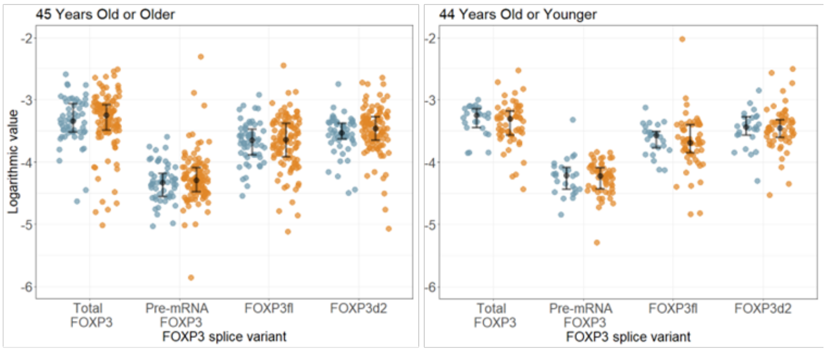

C– KTRs, first day post-transplant

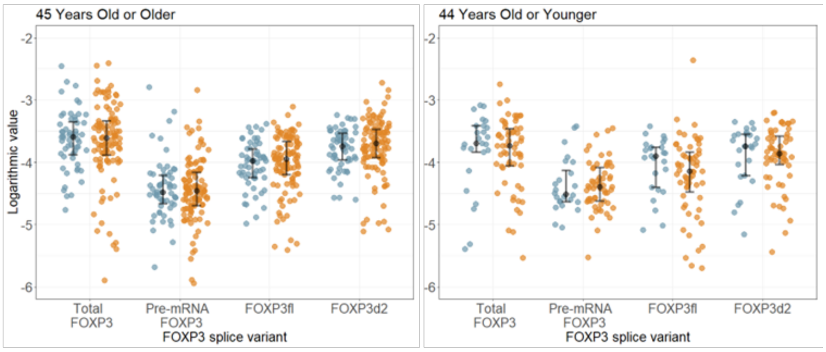

D – KTRs 29 days post-transplant

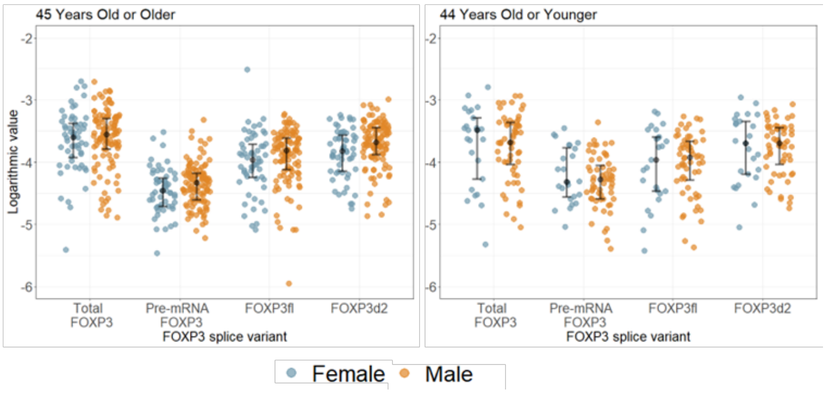

Supplementary Figure 2 - Fold difference of pre-transplant FOXP3 transcript levels in kidney transplant recipients relative to healthy kidney donor levels (mean indicated by dashed line) in A) all included kidney transplant recipients, B) female kidney transplant recipients, and male kidney transplant recipients.

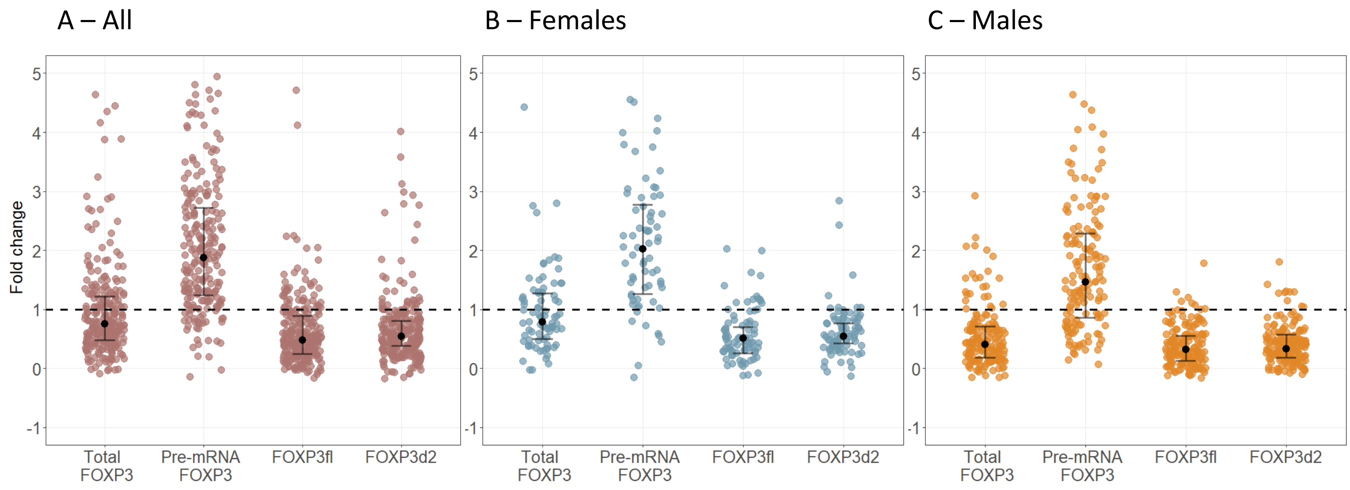

Supplement: Supplementary file 1 — Supplementary Information. [file 41598_2024_62149_MOESM1_ESM.pdf]
